# Blood–Brain Barrier: Structure, Function, Diseases, and Drug Delivery Systems

**DOI:** 10.1002/mco2.70712

**Published:** 2026-04-01

**Authors:** Yanan He, Mengyao Qu, Lu Yu, Lipeng He, Yixun Lu, Jin Hong, Miao Sun, Huikai Yang, Weidong Mi, Hang Guo, Yulong Ma

**Affiliations:** ^1^ Department of Anesthesiology The First Medical Center of Chinese PLA General Hospital Beijing China; ^2^ School of Chemistry and Materials Science Anhui Normal University Wuhu China; ^3^ Department of Anesthesiology The Seventh Medical Center of Chinese PLA General Hospital Beijing China; ^4^ The Second School of Clinical Medicine Southern Medical University Guangzhou China

**Keywords:** blood–brain barrier, drug delivery systems, ischemic stroke, nanoparticles, neurological diseases, neurovascular unit, targeted therapy

## Abstract

The blood–brain barrier (BBB) is a highly selective and dynamic neurovascular interface essential for maintaining central nervous system homeostasis. This specialized barrier comprises brain microvascular endothelial cells interconnected by tight junctions, supported by pericytes and astrocytic end‐feet within the neurovascular unit. While protecting the brain from circulating pathogens and toxins, the BBB presents formidable obstacles to drug delivery, restricting approximately 98% of small‐molecule therapeutics and nearly all large biomolecules from reaching the brain parenchyma. BBB dysfunction is critically implicated in the pathogenesis and progression of numerous neurological disorders, including ischemic stroke, Alzheimer's disease, Parkinson's disease, multiple sclerosis, and brain tumors. This comprehensive review systematically examines the structural organization and functional characteristics of the BBB, elucidates its pathophysiological roles across major neurological diseases, and critically evaluates innovative drug delivery strategies designed to overcome this biological barrier. We analyze passive targeting approaches, active targeting mechanisms via receptor‐mediated transcytosis, and stimuli‐responsive systems including focused ultrasound and magnetic guidance. Additionally, we discuss multifunctional nanoplatforms, biomimetic cell membrane‐coated delivery systems, current preclinical evidence, and clinical translation challenges. Finally, we propose future research directions and identify specific experimental pathways to accelerate the development of next‐generation BBB‐targeted therapeutics from preclinical promise to clinical application.

## Graphical abstract

This graphical abstract illustrates how blood–brain barrier‐targeted nanomedicine integrates diverse nanocarrier platforms with mechanism‐based therapeutic strategies to overcome delivery barriers in ischemic stroke, enabling targeted modulation of oxidative stress, inflammation, excitotoxicity, and neuronal survival.

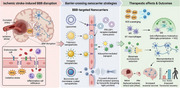

## 1 Introduction

The blood–brain barrier (BBB) represents one of the most sophisticated and selective biological interfaces in the human body, serving as a critical gatekeeper of central nervous system (CNS) homeostasis [1, 2, 3]. The concept of a barrier separating blood from brain tissue was first proposed by Paul Ehrlich in the late 19th century, when he observed that intravenously injected dyes stained peripheral organs but not the brain. This observation was subsequently confirmed and extended by Edwin Goldmann's seminal experiments in the early 20th century, which demonstrated that dyes injected directly into the cerebrospinal fluid stained the brain but not peripheral tissues [4]. These pioneering studies established the foundation for our modern understanding of the BBB. Structurally, the BBB comprises brain microvascular endothelial cells (BMECs) interconnected by elaborate tight junction complexes, forming a continuous, nonfenestrated endothelium with extremely low paracellular permeability [5, 6]. These endothelial cells are supported by pericytes, which share a common basement membrane (BM), and astrocytic end‐feet that ensheath approximately 99% of the cerebrovascular surface, collectively forming the neurovascular unit (NVU) [7, 8]. This intricate cellular architecture, combined with specialized transport systems and metabolic enzymes, enables the BBB to precisely regulate molecular exchange between blood and brain parenchyma, while simultaneously restricting approximately 98% of small‐molecule therapeutics and virtually all macromolecular drugs from entering the CNS [9].

The past decade has witnessed transformative advances in our understanding of blood‐brain barrier (BBB) biology and pathophysiology. Contemporary research has established that the BBB functions not as a static physical barrier, but rather as a dynamic, responsive interface that actively regulates molecular transport, immune surveillance, and neurovascular coupling [10, 11]. The identification of specific transport mechanisms, including receptor‐mediated transcytosis (RMT) via transferrin (Tf) receptor (TfR), low‐density lipoprotein receptor‐related protein 1 (LRP1), and lactoferrin (Lf) receptor (LfR), as well as carrier‐mediated transport (CMT) systems for essential nutrients, has opened promising therapeutic avenues for CNS drug delivery [12]. Importantly, BBB dysfunction has been increasingly recognized as a critical contributor to the pathogenesis and progression of numerous neurological disorders, including ischemic stroke, Alzheimer's disease (AD), Parkinson's disease (PD), multiple sclerosis (MS), and primary brain tumors, positioning this barrier as both a compelling therapeutic target and a significant delivery challenge [13, 14, 15].

Despite remarkable progress in neuroscience and pharmaceutical sciences, effective treatment of CNS disorders remains severely hampered by the BBB's inherently restrictive nature. The clinical failure of numerous promising neuroprotective agents, largely attributed to inadequate brain penetration, underscores the urgent and unmet need for innovative delivery strategies [16]. Recent advances in nanotechnology have yielded diverse approaches to overcome BBB limitations, including polymeric nanoparticles, liposomes, dendrimers, inorganic nanoparticles, and biomimetic systems [17]. These nanomaterial‐based drug delivery systems offer unique advantages through passive targeting strategies (size optimization, surface modification with polyethylene glycol [PEG]), active targeting mechanisms (RMT via Tf, Lf, and peptide conjugation), and stimuli‐responsive systems (focused ultrasound [FUS], magnetic field guidance, pH‐sensitive release) [18, 19, 20].

Notably, while most existing reviews organize nanotherapeutics by carrier type (liposomes, polymeric nanoparticles, inorganic nanoparticles), this approach often obscures the therapeutic rationale underlying nanocarrier selection for specific pathological targets. In contrast, our review adopts a mechanism‐based classification framework that organizes nanotherapeutics according to their primary therapeutic mechanisms—reactive oxygen species (ROS)‐scavenging, glutamate‐modulating, anti‐inflammatory, neuroprotective/neuroregenerative, and multifunctional platforms—thereby providing clearer guidance for rational therapeutic design matched to specific stages of disease pathophysiology. Furthermore, this review integrates the most recent advances in biomimetic delivery systems, including cell membrane‐coated nanoparticles and exosome‐based carriers, as well as emerging mRNA‐loaded nanoplatforms for neurological applications (2024–2025), which represent paradigm shifts in the field that have not been comprehensively addressed in previous reviews.

This comprehensive review systematically examines the BBB from structural, functional, pathological, and therapeutic perspectives. We first delineate the anatomical and molecular organization of the BBB, including tight junction architecture, transport mechanisms, and NVU components. Subsequently, we analyze BBB dysfunction across major neurological diseases, encompassing ischemic stroke, neurodegenerative disorders, and brain tumors. The review then critically evaluates current nanomaterial‐based drug delivery strategies, comparing passive and active targeting approaches, stimuli‐responsive systems, and emerging biomimetic platforms. Finally, we discuss clinical translation challenges, current limitations, and future directions. Through this integrated framework, we aim to provide researchers and clinicians with comprehensive insights and actionable guidance for developing next‐generation BBB‐targeted therapeutics.

## 2 Structure of the BBB

**FIGURE 1:**
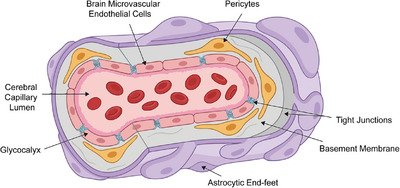
Structural organization of the blood–brain barrier (BBB). The cross‐sectional schematic identifies the complex neurovascular unit. Moving outward from the cerebral capillary lumen, the structure comprises brain microvascular endothelial cells (pink) lined with glycocalyx and sealed by tight junctions (blue). These are supported by the basement membrane, pericytes (yellow), and the outermost astrocytic end‐feet (purple), which completely ensheath the vessel. The figure also depicts peripheral immune cell interactions at the neurovascular interface.

The BBB represents a complex, multicellular structure forming the interface between cerebral vasculature and brain parenchyma. This section systematically examines five key structural components: BMECs, tight junctions, pericytes, astrocytic end‐feet, and BM. These components function coordinately to establish the BBB's unique barrier properties, with each element contributing distinct but interconnected roles in maintaining CNS homeostasis. Understanding this detailed structural organization is essential for developing effective drug delivery strategies that can exploit specific cellular and molecular features while preserving barrier integrity. Importantly, BBB opening in pathological conditions is spatially heterogeneous (with greater disruption in the ischemic core than the penumbra) and temporally dynamic (biphasic opening pattern over hours to days), complicating drug exposure, dosing optimization, and therapeutic windowing. This heterogeneity motivates the development of targeted delivery systems and on‐demand release strategies that can adapt to regional and temporal variations in barrier permeability (Figure 1).

### 2.1 Brain Microvascular Endothelial Cells

BMECs constitute the primary cellular component of the BBB and exhibit unique characteristics distinguishing them from peripheral endothelium [21, 22, 23]. Unlike their counterparts in other organs, BMECs are connected by exceptionally tight junctions. These cells display remarkably low rates of transcytosis and pinocytosis. Additionally, they lack fenestrations and express high levels of efflux transporters. The luminal surface is covered by a glycocalyx layer composed of proteoglycans, glycoproteins, and glycosaminoglycans, providing additional barrier function and serving as a mechanosensor for blood flow [24]. BMECs express specific transporters for essential nutrients, including glucose transporter 1 (GLUT1) and various amino acid transporters, as well as RMT systems for larger molecules such as Tf and insulin [25, 26, 27]. These transport systems have been extensively exploited for targeted drug delivery, as discussed in Section 5. The unique phenotype of BMECs is not cell‐autonomous but requires continuous signaling from neighboring pericytes and astrocytes, highlighting the integrated nature of the NVU.

### 2.2 Tight Junctions and Molecular Architecture

Tight junctions represent the defining structural feature of the BBB, creating a nearly impermeable paracellular barrier with electrical resistance of approximately 1500–2000 Ω cm^2^ [28, 29]. The molecular architecture comprises transmembrane proteins including claudins (particularly claudin‐5, claudin‐3, and claudin‐12), occludin, and junctional adhesion molecules (JAMs), which interact with cytoplasmic scaffolding proteins such as zonula occludens proteins (ZO‐1, ZO‐2, ZO‐3) [30]. Claudin‐5 is the most abundantly expressed claudin in brain endothelium; knockout studies demonstrate that claudin‐5 deficiency results in size‐selective BBB leakage to molecules up to 800 Da [31]. Occludin plays regulatory roles in barrier function and signaling, while JAMs contribute to TJ assembly and leukocyte transmigration during inflammation [32]. The cytoplasmic scaffolding proteins anchor transmembrane proteins to the actin cytoskeleton, enabling dynamic regulation of barrier permeability. This dynamic nature allows the BBB to respond to physiological demands while maintaining protective function.

### 2.3 Pericytes and Their Role in BBB Maintenance

Pericytes are mural cells embedded within the vascular BM, sharing direct contact with endothelial cells through specialized peg‐socket junctions [33, 34]. The brain microvasculature exhibits the highest pericyte coverage of any vascular bed, with a pericyte‐to‐endothelial cell ratio of approximately 1:1 to 1:3. Pericytes play multifaceted roles: regulating capillary blood flow through contractile processes, contributing to BM synthesis, providing growth factors essential for endothelial survival, and inducing barrier properties in BMECs [35, 36]. Studies in pericyte‐deficient mice demonstrate increased BBB permeability, enhanced transcytosis rates, and abnormal tight junction protein expression [37]. Recent research has identified pericytes as key regulators of neurovascular coupling and implicated their dysfunction in AD and diabetic retinopathy [38, 39]. Pericyte degeneration represents an early pathological event in several neurological disorders, positioning these cells as potential therapeutic targets for BBB restoration strategies.

### 2.4 Astrocytic End‐Feet and the Gliovascular Interface

Astrocytes extend specialized processes called end‐feet that ensheath approximately 99% of the cerebral microvasculature, forming the outermost layer of the BBB [40, 41]. Astrocytic end‐feet express high densities of aquaporin‐4 (AQP4) water channels and potassium channel Kir4.1, playing critical roles in water homeostasis, potassium buffering, and waste clearance through the glymphatic system [42, 43]. Astrocytes secrete factors including sonic hedgehog, angiopoietin‐1, and glial‐derived neurotrophic factor (GDNF) that induce and maintain BBB properties [44]. Furthermore, astrocytes participate in neurovascular coupling by translating neuronal activity into vascular responses, regulating cerebral blood flow according to local metabolic demands [45]. Astrocytic dysfunction or loss of end‐foot polarity has been implicated in BBB breakdown in stroke, AD, and epilepsy [46, 47]. The polarized distribution of AQP4 at end‐feet is essential for glymphatic function; its disruption impairs interstitial solute clearance, contributing to protein aggregate accumulation in neurodegenerative diseases.

### 2.5 BM Components

The BM is a specialized extracellular matrix that surrounds brain capillaries, providing structural support and signaling platforms for BBB cells. The vascular BM comprises two biochemically distinct layers: an inner endothelial BM produced by BMECs and an outer parenchymal BM produced by astrocytes, with pericytes residing between these layers [48, 49]. Major BM components include type IV collagen (particularly α1, α2, α4, and α5 chains), laminins (especially laminin‐411, ‐421, ‐511, and ‐521), nidogens, and heparan sulfate proteoglycans including perlecan and agrin [50, 51, 52]. These components provide mechanical support, anchor adjacent cells through integrin interactions, sequester growth factors, and influence cell survival and differentiation. During pathological conditions, matrix metalloproteinases (MMPs), particularly MMP‐2 and MMP‐9, degrade BM components, contributing to BBB disruption and facilitating immune cell infiltration [53, 54].

In summary, the BBB represents a highly organized yet dynamically regulated interface formed by endothelial tight junctions, transport systems, mural cells, and perivascular support elements. Under pathological conditions such as ischemic stroke, this architecture undergoes spatially and temporally heterogeneous alterations, leading to region‐ and time‐dependent changes in permeability. These features simultaneously restrict uniform drug access while creating transient therapeutic opportunities. A clear structural and functional understanding of the BBB therefore provides the essential foundation for rational design of delivery strategies, setting the stage for the discussion of BBB penetration mechanisms in the following section.

## 3 Physiological Functions of the BBB

**FIGURE 2:**
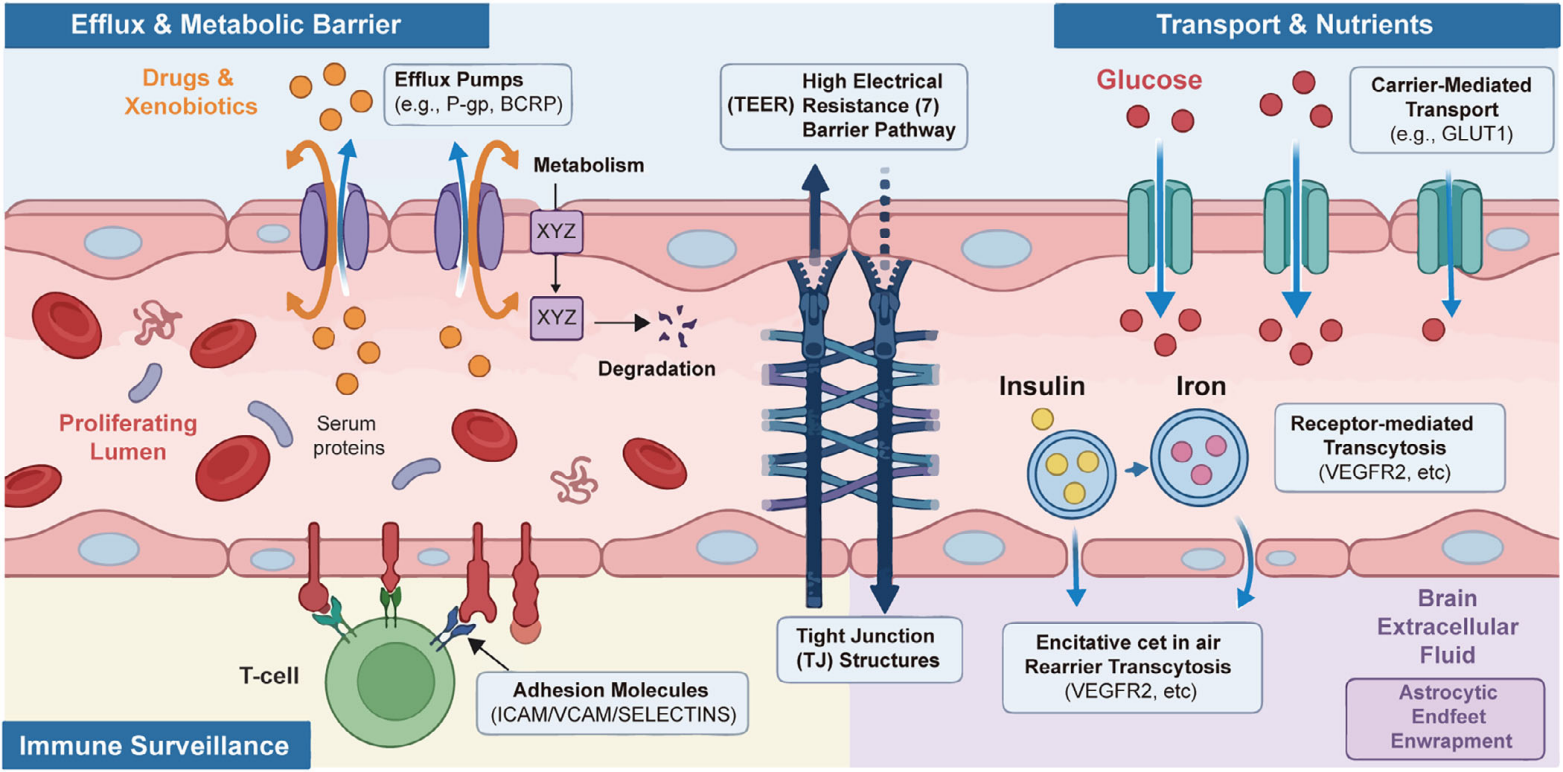
Schematic overview of blood–brain barrier (BBB) physiological functions. This diagram illustrates the BBB biological interface maintaining CNS homeostasis through four synergistic protection mechanisms: (1) a stringent physical and metabolic barrier formed by tight junction complexes and efflux transporters (e.g., P‐gp pumps) that actively prevent neurotoxin accumulation; (2) precise ionic and neurotransmitter homeostasis maintained through active transport mechanisms (e.g., Na^+^/K^+^‐ATPase, excitatory amino acid transporters) that ensure a stable neuronal microenvironment; (3) highly selective carrier‐mediated and receptor‐mediated transport systems for nutrient delivery (e.g., glucose, macromolecules); and (4) a controlled neuroimmune interface that regulates immune surveillance via specific endothelial adhesion molecules. Multifunctional/biomimetic platforms can engage multiple mechanisms concurrently (e.g., ROS‐scavenging + anti‐inflammatory modulation + neurotrophic support).

The BBB serves multiple critical physiological functions that collectively maintain the optimal microenvironment required for proper neuronal function. Beyond its role as a simple physical barrier, the BBB actively regulates the bidirectional transport of nutrients, metabolic waste products, and signaling molecules between the blood and brain parenchyma, while simultaneously providing immune surveillance and protection against circulating pathogens and neurotoxic substances. This section systematically examines four fundamental functional domains of the BBB: physical and metabolic barrier functions, transport systems for nutrient delivery, efflux transporter‐mediated brain protection, and the neuroimmune interface. Understanding these physiological functions is essential not only for appreciating how the BBB maintains CNS homeostasis, but also for identifying therapeutic windows and rational drug delivery strategies that can exploit or circumvent specific barrier mechanisms (Figure 2).

### 3.1 Physical and Metabolic Barrier Functions

The physical barrier function of the BBB is primarily mediated by tight junction complexes that seal adjacent BMECs. These complexes create an extremely high transendothelial electrical resistance of approximately 1500–2000 Ω cm^2^. This resistance is substantially higher than peripheral endothelia (3–33 Ω cm^2^). This remarkable tightness is achieved through coordinated expression of transmembrane proteins including claudins (particularly claudin‐5), occludin, and JAMs, which are anchored to the actin cytoskeleton via cytoplasmic scaffolding proteins such as zonula occludens (ZO‐1, ZO‐2, ZO‐3) [55, 56, 57]. Complementing the physical barrier, the metabolic barrier function is mediated by diverse intracellular and extracellular enzymes, including cytochrome P450 enzymes, monoamine oxidases (MAO‐A and MAO‐B), γ‐glutamyl transpeptidase, and various peptidases that metabolize drugs and neurotransmitters before they reach the brain parenchyma [58, 59, 60]. Furthermore, efflux transporters of the ATP‐binding cassette (ABC) family, particularly P‐glycoprotein (P‐gp/ABCB1), breast cancer resistance protein (BCRP/ABCG2), and multidrug resistance‐associated proteins (MRPs/ABCCs), actively pump lipophilic compounds back into the bloodstream, significantly limiting brain accumulation of many therapeutic agents [61, 62]. The coordinated action of these physical and metabolic barriers creates a highly selective interface that protects the CNS from potentially harmful substances while permitting essential molecular exchange.

### 3.2 Ion Homeostasis and Neurotransmitter Regulation

Precise regulation of the ionic microenvironment is essential for proper neuronal excitability and synaptic transmission, and the BBB plays a central role in maintaining this homeostasis [63]. Unlike peripheral tissues where interstitial ion concentrations fluctuate with plasma levels, the brain extracellular fluid maintains remarkably stable concentrations of K^+^, Na^+^, Ca^2^^+^, and Mg^2^^+^ through active transport mechanisms at the BBB [64]. The Na^+^/K^+^‐ATPase pump, located predominantly on the abluminal membrane of BMECs, actively maintains low extracellular K^+^ concentrations (approximately 2.5–3.0 mM) critical for neuronal resting membrane potential and action potential generation [65, 66]. The BBB also critically regulates neurotransmitter homeostasis by preventing circulating neurotransmitters from entering the brain while facilitating the removal of excess neurotransmitters from the CNS through specific transport systems, including excitatory amino acid transporters (EAATs) for glutamate and GABA transporters [67, 68]. This regulation is particularly important for glutamate, as the BBB expresses high levels of EAAT1 and EAAT2, which efficiently clear glutamate from the brain interstitium, thereby preventing excitotoxic neuronal death under physiological conditions.

### 3.3 Transport Systems and Nutrient Delivery

Despite its restrictive nature, the BBB must facilitate the delivery of essential nutrients to meet the high metabolic demands of the brain, which consumes approximately 20% of the body's glucose and oxygen despite representing only 2% of body weight [69, 70]. This selective permeability is achieved through specialized transport systems including CMT, RMT, and adsorptive‐mediated transcytosis (AMT) [3]. Glucose transporter 1 (GLUT1/SLC2A1), highly expressed on both luminal and abluminal membranes of BMECs, mediates the facilitated diffusion of glucose into the brain, while large neutral amino acid transporter 1 (LAT1/SLC7A5) transports essential amino acids including phenylalanine, tyrosine, leucine, and tryptophan, which serve as precursors for neurotransmitter synthesis [71, 72, 73]. Monocarboxylate transporters (MCTs), particularly MCT1, facilitate the transport of lactate, pyruvate, and ketone bodies, providing alternative energy substrates during glucose deprivation or ketogenic states [74, 75]. RMT enables the transport of larger molecules, including proteins and peptides, through specific receptor–ligand interactions involving the TfR for iron delivery, insulin receptor for insulin transport, and low‐density LRP1 for various ligands including apolipoprotein E (APOE) and amyloid‐β (Aβ) [76]. These RMT pathways have been extensively exploited for brain‐targeted drug delivery strategies.

### 3.4 Immune Privilege and Neuroimmune Interface

The BBB plays a pivotal role in regulating immune cell trafficking and maintaining the immune‐privileged status of the CNS [77]. Under physiological conditions, the BBB strictly limits the entry of peripheral immune cells into the brain parenchyma, with only small numbers of activated T lymphocytes conducting routine immunosurveillance, achieved through low expression of leukocyte adhesion molecules on resting BMECs and the presence of tight junctions that prevent paracellular immune cell migration [78]. However, the BBB is not immunologically inert but actively participates in neuroimmune communication by expressing pattern recognition receptors, including Toll‐like receptors, enabling detection and response to pathogen‐associated molecular patterns and damage‐associated molecular patterns [79, 80]. Upon activation, BMECs can upregulate adhesion molecules such as intercellular adhesion molecule‐1 (ICAM‐1), vascular cell adhesion molecule‐1 (VCAM‐1), and E‐selectin, facilitating leukocyte adhesion and transmigration during neuroinflammatory conditions, while also secreting various cytokines and chemokines that modulate both peripheral and central immune responses.

Taken together, the BBB maintains CNS homeostasis through the coordinated interplay of physical restriction, metabolic degradation, selective nutrient transport, ionic buffering, and regulated immune surveillance. These physiological functions not only protect the brain from harmful substances but also create a tightly controlled microenvironment essential for neuronal signaling and synaptic function. Importantly, each of these functional domains presents potential vulnerabilities that can be exploited—or disrupted—under pathological conditions. Understanding the precise mechanisms underlying these functions therefore provides the necessary foundation for appreciating how BBB dysfunction contributes to neurological diseases, as discussed in the following section.

## 4 BBB Dysfunction in Neurological Diseases

**FIGURE 3:**
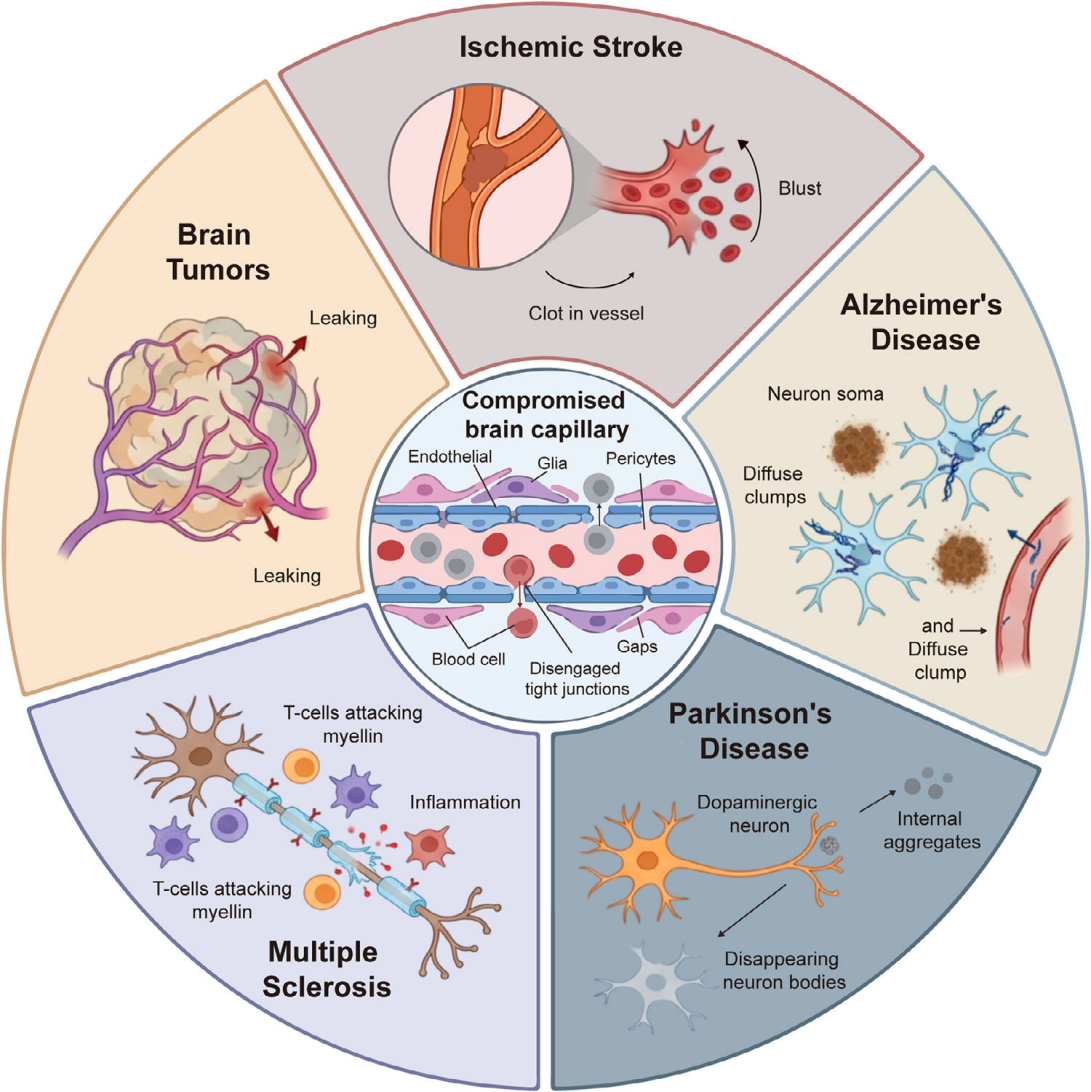
Blood–brain barrier dysfunction in neurological diseases. Central panel illustrates the compromised brain capillary structure, showing endothelial cells, glia, pericytes, blood cells, and disengaged tight junctions with gaps. Surrounding panels depict disease‐specific BBB pathology: ischemic stroke—vessel occlusion by clot leading to blood cell extravasation; Alzheimer's disease—neuronal degeneration with diffuse amyloid clumps and vascular amyloid deposition; Parkinson's disease—dopaminergic neuron loss with intracellular aggregates and disappearing neuron bodies; multiple sclerosis—T‐cell‐mediated myelin attack and neuroinflammation; brain tumors—abnormal vasculature with increased permeability and leakage.

BBB dysfunction is increasingly recognized as both a consequence and contributing factor in numerous neurological disorders, playing a pivotal role in disease initiation, progression, and therapeutic outcomes. This section examines BBB dysfunction across five disease categories: (i) ischemic stroke, (ii) AD, (iii) PD, (iv) MS, and (v) brain tumors. Compromised BBB integrity can initiate or exacerbate pathological processes through various mechanisms, including infiltration of blood‐derived proteins and immune cells, disruption of ionic and metabolic homeostasis, impaired clearance of neurotoxic substances, and aberrant neurovascular coupling [81]. Emerging evidence demonstrates that BBB breakdown is not merely a secondary consequence of neurological injury but actively participates in disease pathogenesis, creating a vicious cycle of neuroinflammation, oxidative stress, and progressive neuronal damage [82]. Understanding disease‐specific BBB alterations is essential for developing targeted therapeutic strategies that can restore barrier integrity, modulate pathological transport, or exploit BBB dysfunction for enhanced drug delivery. In this section, we systematically examine BBB dysfunction in major neurological diseases, including ischemic stroke, neurodegenerative disorders (AD, PD), MS, and brain tumors, highlighting the distinct pathophysiological mechanisms and therapeutic implications in each condition (Figure 3).

### 4.1 Ischemic Stroke

Ischemic stroke, caused by arterial occlusion leading to cerebral hypoperfusion, represents one of the most dramatic examples of acute BBB dysfunction and remains a leading cause of death and disability worldwide [83, 84]. The pathophysiology of BBB disruption in ischemic stroke follows a biphasic temporal pattern, with an initial opening occurring within hours of ischemia onset, followed by a secondary delayed disruption that can persist for days to weeks [85]. Importantly, BBB opening is heterogeneous, with the ischemic core exhibiting more severe and earlier disruption compared with the penumbral region, which has significant implications for therapeutic intervention windows [86, 87]. During the acute phase, cerebral ischemia triggers a cascade of pathological events including energy depletion, ionic imbalance, excitotoxicity, oxidative stress, and inflammation, all of which converge to compromise BBB integrity [88, 89]. The initial reduction in cerebral blood flow leads to ATP depletion and failure of energy‐dependent ion pumps, resulting in cytotoxic edema and disruption of tight junction proteins, particularly claudin‐5, occludin, and ZO‐1 [90, 91]. MMPs, especially MMP‐2 and MMP‐9, are rapidly upregulated and activated following ischemia, degrading tight junction proteins and BM components, thereby facilitating BBB breakdown [92, 93]. The resulting increase in BBB permeability allows extravasation of plasma proteins, including albumin, fibrinogen, and immunoglobulins, into the brain parenchyma, triggering vasogenic edema, increased intracranial pressure, and secondary neuronal injury [94, 95]. Furthermore, the compromised BBB permits infiltration of peripheral immune cells, including neutrophils, monocytes, and lymphocytes, which contribute to postischemic neuroinflammation through release of proinflammatory cytokines, ROS, and proteolytic enzymes [96, 97]. The secondary phase of BBB disruption, occurring 48–72 h poststroke, is associated with reperfusion injury, sustained inflammation, and angiogenic remodeling [98]. The extent and duration of BBB dysfunction correlate with stroke severity, infarct volume, and clinical outcomes, making BBB integrity a critical therapeutic target and prognostic biomarker that can be assessed using dynamic contrast‐enhanced magnetic resonance imaging (DCE‐MRI) permeability mapping and serum markers such as interleukin‐6 (IL‐6) and MMP‐9.

### 4.2 Alzheimer's Disease

AD, the most common neurodegenerative disorder, is characterized by progressive cognitive decline, accumulation of Aβ plaques, neurofibrillary tangles composed of hyperphosphorylated tau protein, and extensive neuronal loss [99, 100]. Accumulating evidence over the past decade has established BBB dysfunction as an early and critical contributor to AD pathogenesis, occurring before significant cognitive impairment and independent of Aβ and tau pathology [101, 102]. Postmortem studies and advanced neuroimaging techniques, including DCE‐MRI, have revealed increased BBB permeability in AD patients, particularly in the hippocampus and medial temporal lobe—regions critical for memory formation and early affected in AD [103, 104]. At the cellular level, AD‐associated BBB dysfunction manifests as pericyte degeneration, reduced tight junction protein expression, BM thickening, and endothelial cell activation [105]. Pericyte loss, which can be detected in early AD stages, leads to compromised BBB integrity, reduced cerebral blood flow, and impaired neurovascular coupling, contributing to chronic cerebral hypoperfusion and metabolic stress [106, 107]. The dysfunctional BBB in AD exhibits impaired clearance of Aβ from the brain, primarily through reduced expression and function of low‐density LRP1, a major Aβ efflux transporter, and increased expression of receptor for advanced glycation end products, which mediates Aβ influx into the brain [108, 109]. This imbalance between Aβ production, aggregation, and clearance promotes cerebral Aβ accumulation and plaque formation [110]. Additionally, BBB breakdown allows infiltration of blood‐derived proteins such as fibrinogen, thrombin, and immunoglobulins, which can directly activate microglia, trigger neuroinflammation, and contribute to synaptic dysfunction and neuronal loss [111, 112]. Recent studies have identified APOE4, the strongest genetic risk factor for late‐onset AD, as a key mediator of BBB dysfunction through activation of the cyclophilin A–MMP‐9 pathway in pericytes, providing a mechanistic link between genetic risk and vascular pathology in AD.

### 4.3 Parkinson's Disease

PD, the second most common neurodegenerative disorder, is characterized by progressive loss of dopaminergic neurons in the substantia nigra pars compacta, accumulation of α‐synuclein aggregates (Lewy bodies), and motor symptoms including tremor, rigidity, and bradykinesia [113]. While traditionally viewed as a primary neuronal disorder, emerging evidence implicates BBB dysfunction and neurovascular pathology in PD pathogenesis and progression [114, 115]. Postmortem studies have revealed structural abnormalities in brain microvessels of PD patients, including reduced capillary density, BM thickening, endothelial cell degeneration, and pericyte loss in the substantia nigra and striatum [116, 117]. Functional BBB disruption in PD has been demonstrated through increased permeability to small molecules and proteins, as evidenced by extravasation of serum proteins including albumin and immunoglobulin G into the brain parenchyma [118, 119]. Advanced neuroimaging studies using DCE‐MRI have confirmed increased BBB permeability in PD patients, particularly in the basal ganglia and substantia nigra, correlating with disease severity and motor dysfunction [120, 121]. At the molecular level, PD‐associated BBB dysfunction involves downregulation of tight junction proteins (claudin‐5, occludin, ZO‐1), increased expression of adhesion molecules (ICAM‐1, VCAM‐1), and activation of MMPs, particularly MMP‐2 and MMP‐9 [122, 123]. Importantly, α‐synuclein, the pathological hallmark protein of PD, has been shown to directly compromise BBB integrity by disrupting tight junctions, inducing endothelial cell dysfunction, and promoting neuroinflammation [124, 125]. The dysfunctional BBB in PD permits infiltration of peripheral immune cells, including T lymphocytes and monocytes, which contribute to chronic neuroinflammation and progressive dopaminergic neurodegeneration [126, 127]. Furthermore, BBB dysfunction impairs the clearance of toxic metabolites and α‐synuclein oligomers from the brain, potentially accelerating disease progression [128]. The compromised BBB also affects the delivery of levodopa, the gold‐standard PD treatment, as competition with large neutral amino acids at the LAT1 can reduce its brain uptake, contributing to motor fluctuations and treatment complications in advanced PD [129].

### 4.4 Multiple Sclerosis

MS is a chronic autoimmune inflammatory disease of the CNS characterized by demyelination, axonal damage, and progressive neurological disability [130, 131]. BBB dysfunction is a cardinal feature and critical initiating event in MS pathogenesis, facilitating the entry of autoreactive T lymphocytes and other immune cells into the CNS, where they orchestrate inflammatory demyelination [132, 133]. In MS, BBB disruption precedes clinical symptoms and demyelinating lesion formation, as demonstrated by gadolinium enhancement on MRI, which reflects areas of active BBB breakdown and ongoing inflammation [134, 135]. The pathophysiology of BBB dysfunction in MS involves multiple mechanisms, including upregulation of endothelial adhesion molecules (E‐selectin, ICAM‐1, VCAM‐1), increased expression of chemokines (C–C motif chemokine ligand 2, C–X–C motif chemokine ligand 12) that attract immune cells, and disruption of tight junction proteins [136, 137]. Proinflammatory cytokines, particularly tumor necrosis factor‐α (TNF‐α), interferon‐γ, and IL‐17, produced by activated T cells and other immune cells, directly compromise BBB integrity by inducing endothelial cell activation, tight junction disassembly, and increased transcytosis [138, 139]. MMPs, especially MMP‐2 and MMP‐9, are elevated in MS lesions and cerebrospinal fluid, contributing to BM degradation and facilitating immune cell transmigration across the BBB [140, 141]. The compromised BBB in MS allows infiltration of CD4+ and CD8+ T lymphocytes, B cells, monocytes, and dendritic cells into the CNS, where they recognize myelin antigens presented by local antigen‐presenting cells, triggering inflammatory cascades that lead to oligodendrocyte death, demyelination, and axonal injury [142, 143]. Additionally, BBB dysfunction permits entry of circulating antibodies and complement proteins, which can directly damage myelin and oligodendrocytes [144]. Importantly, BBB integrity partially recovers during remission phases in relapsing‐remitting MS, but chronic BBB dysfunction persists in progressive MS forms, contributing to ongoing neurodegeneration and disability accumulation [145, 146].

### 4.5 Brain Tumors

Brain tumors, including primary malignancies such as glioblastoma multiforme (GBM) and metastatic brain tumors, present unique challenges related to BBB dysfunction and the blood–tumor barrier (BTB) [147, 148]. Unlike the intact BBB, the BTB exhibits heterogeneous and compromised barrier function, characterized by increased permeability, abnormal angiogenesis, and altered expression of transporters and receptors [149, 150]. In high‐grade gliomas, particularly GBM, the tumor microenvironment drives extensive angiogenesis through secretion of vascular endothelial growth factor (VEGF) and other proangiogenic factors, resulting in the formation of structurally and functionally abnormal blood vessels [151, 152]. These tumor‐associated vessels exhibit irregular architecture, discontinuous endothelium, reduced pericyte coverage, fragmented BM, and disrupted tight junctions, leading to increased permeability and vasogenic edema [153, 154]. However, BTB permeability is highly heterogeneous within individual tumors, with the tumor core often showing greater barrier disruption than the infiltrative tumor margin, where relatively intact BBB can limit drug penetration [155, 156]. This heterogeneity poses significant challenges for therapeutic drug delivery, as regions with intact BBB restrict chemotherapeutic agent accumulation, contributing to treatment resistance and tumor recurrence [157]. Furthermore, tumor‐associated endothelial cells maintain expression of efflux transporters, particularly P‐gp and BCRP, which actively pump chemotherapeutic agents back into the bloodstream, further limiting drug efficacy [158]. Metastatic brain tumors, originating from primary cancers in the lung, breast, melanoma, and other organs, also exhibit variable BTB dysfunction depending on tumor type, size, and location [159]. Interestingly, micrometastases and small metastatic lesions may be surrounded by relatively intact BBB, preventing adequate drug delivery and contributing to treatment failure [160]. Recent studies have revealed that tumor cells can actively modulate BTB permeability through secretion of VEGF, MMPs, and inflammatory mediators, creating a permissive microenvironment for tumor growth and invasion [161, 162]. Understanding the complex and dynamic nature of BTB dysfunction is critical for developing effective drug delivery strategies and improving therapeutic outcomes in brain tumor patients [163, 164].

In summary, BBB dysfunction is a shared yet mechanistically distinct feature across the neurological diseases discussed above. In ischemic stroke, BBB disruption is acute and biphasic; in AD and PD, it develops insidiously as an early pathological event; in MS, it serves as a gateway for autoimmune attack; and in brain tumors, the BTB exhibits heterogeneous and tumor‐modulated permeability. Recognizing these disease‐specific patterns of BBB alteration is essential for selecting appropriate therapeutic strategies—whether aimed at restoring barrier integrity, modulating pathological transport, or exploiting transient permeability windows for enhanced drug delivery. These pathological insights directly inform the design of BBB‐targeted delivery systems, which are discussed in the following section.

## 5 Strategies for BBB Penetration and Brain Targeting

**FIGURE 4:**
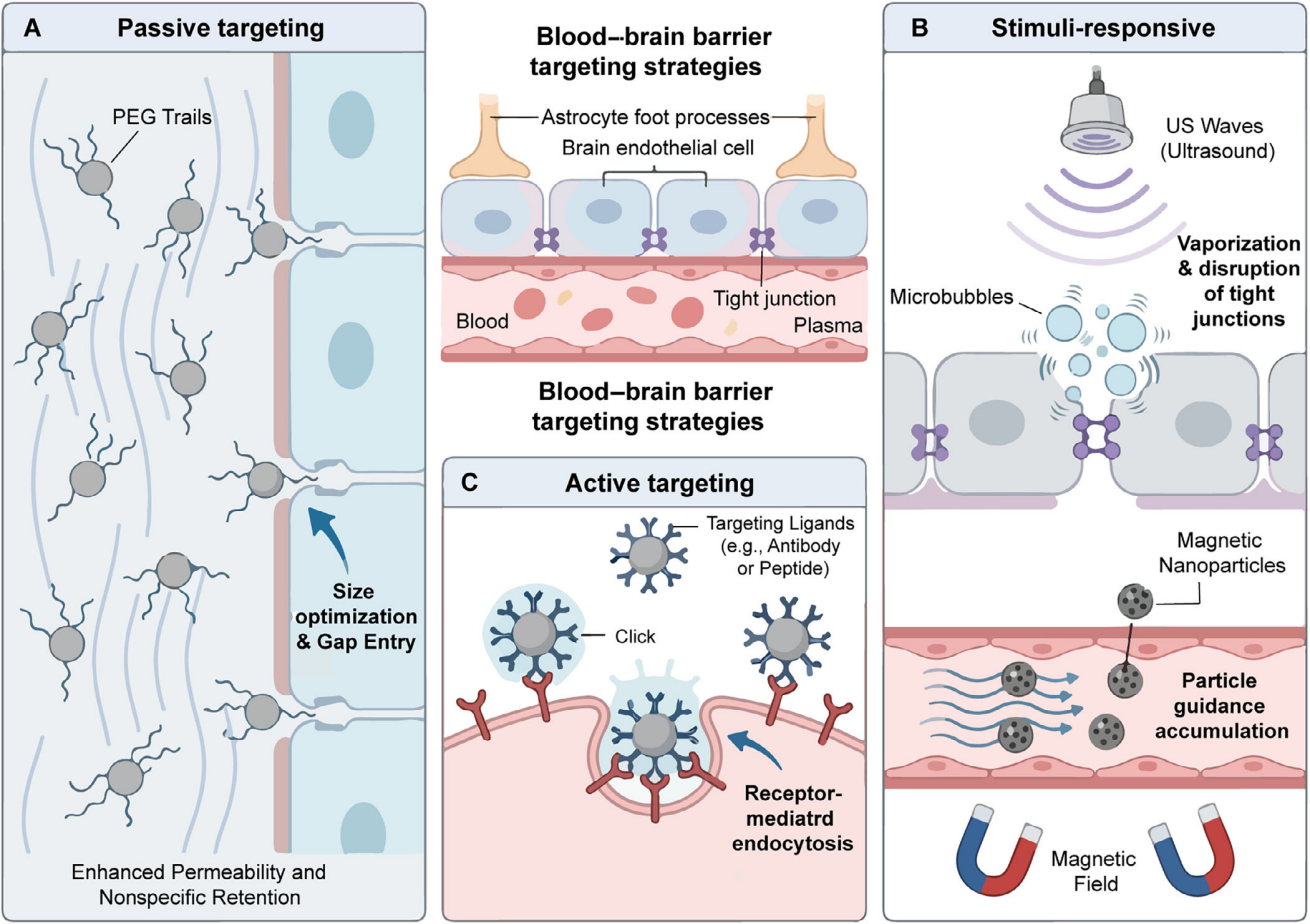
Schematic illustration of nanotechnology‐based strategies for crossing the blood–brain barrier (BBB). The diagram depicts three principal targeting mechanisms to overcome BBB impermeability: (A) passive targeting, which relies on physiochemical optimization (e.g., size reduction) and surface PEGylation to prolong circulation and exploit nonspecific retention gaps; (B) stimuli‐responsive strategies, employing external physical triggers such as ultrasound waves (causing microbubble oscillations to disrupt tight junctions) or magnetic fields to guide magnetic carrier accumulation; and (C) active targeting, where nanoparticles functionalized with specific surface ligands (e.g., antibodies or peptides) bind to endothelial receptors, inducing receptor‐mediated endocytosis for specific transmembrane transport.

The development of effective strategies to overcome the BBB represents a critical challenge in CNS drug delivery, as the restrictive nature of the BBB limits the brain penetration of approximately 98% of small‐molecule drugs and virtually all macromolecular therapeutics [165, 166, 167]. Over the past decade, significant advances in nanotechnology, molecular biology, and biomedical engineering have yielded diverse approaches to enhance drug delivery across the BBB, ranging from physicochemical optimization of drug molecules to sophisticated nanocarrier systems and external stimuli‐responsive platforms [168, 169, 170]. These strategies can be broadly categorized into passive targeting approaches that exploit physicochemical properties and the enhanced permeability and retention (EPR) effect, active targeting mechanisms utilizing RMT and CMT, and stimuli‐responsive systems enabling spatiotemporal control of drug release and BBB permeability modulation [171, 172]. Understanding the principles, advantages, and limitations of each approach is essential for rational design of brain‐targeted drug delivery systems. In this section, we provide a comprehensive analysis of current BBB penetration strategies, highlighting recent advances, comparative efficacy, and clinical translation potential (Figure 4).

### 5.1 Passive Targeting: Size Optimization and Surface Modification

Passive targeting strategies leverage the optimization of nanoparticle physicochemical properties to enhance BBB penetration and cerebral accumulation, exploiting stroke‐induced BBB disruption through mechanisms including the EPR‐like effect and AMT [173, 174]. Size optimization constitutes a critical determinant of BBB penetration efficiency. Nanoparticles within the 10–100 nm range exhibit superior cerebral uptake, as this dimensional window enables exploitation of stroke‐induced endothelial tight junction disruption and paracellular transport pathways while circumventing rapid opsonization and subsequent clearance by the mononuclear phagocyte system [175].

It should be noted that the EPR effect in brain ischemia is considerably less reliable than in solid tumors due to the heterogeneous and time‐dependent nature of BBB disruption following stroke [176, 177]. The formation of a protein corona upon nanoparticle exposure to biological fluids significantly influences biodistribution and cellular uptake, and must be considered in nanoparticle design [178, 179]. Factors including size, polydispersity index (PDI), and surface charge require careful optimization to ensure reproducible targeting efficiency. Recent studies emphasize the importance of maintaining PDI below 0.2 for predictable pharmacokinetic profiles [180].

Surface PEGylation remains a cornerstone strategy for pharmacokinetic enhancement, whereby PEG chains form a hydrophilic corona that sterically inhibits opsonization and phagocytic clearance. This “stealth” modification prolongs systemic circulation, consequently augmenting nanoparticle accumulation at the BBB interface and facilitating transcytotic penetration into the cerebral parenchyma. However, emerging concerns regarding accelerated blood clearance phenomenon upon repeated administration and the development of anti‐PEG antibodies have prompted researchers to explore alternative hydrophilic polymers. Poly(2‐oxazoline), polysarcosine, and zwitterionic polymers have emerged as promising next‐generation stealth coatings that offer comparable or superior circulation extension without eliciting anti‐PEG immune responses.

Surface charge modulation constitutes another pivotal parameter in passive BBB targeting, given that nanoparticle surface charge profoundly governs electrostatic interactions with the negatively charged glycocalyx and tight junction proteins of brain capillary endothelial cells. Zwitterionic surface modifications combining both positive and negative charges have shown promise in achieving ultra‐low fouling properties while maintaining sufficient BBB interaction capabilities. Charge‐reversible nanoparticles that maintain neutral charge in systemic circulation but undergo conversion to positive charge in the acidic ischemic microenvironment represent an elegant solution to balance circulation longevity with cellular uptake efficiency. To facilitate direct comparison across nanocarrier platforms with respect to BBB penetration mechanisms, advantages, limitations, and translational potential, the major nanocarrier systems investigated for ischemic stroke are summarized in Table 1.

**TABLE 1:** Comparative overview of major nanocarrier platforms for BBB‐targeted drug delivery in ischemic stroke.

Nanocarrier type	Typical composition	Size range (nm)	BBB‐crossing mechanisms	Key advantages	Major limitations	Representative stroke models and outcomes	References
Liposomes	Phospholipids, cholesterol	80–150	Passive diffusion; receptor‐mediated transcytosis (TfR/LRP1); FUS‐assisted	Excellent biocompatibility; high drug loading; clinical precedent	Limited stability; premature drug leakage	MCAO rat, i.v., 2 h postreperfusion: ↓ infarct volume ∼30–40%, ↑ mNSS	[17, 181, 182]
Polymeric nanoparticles	PLGA, PEG–PLGA	50–200	Passive targeting; ligand‐mediated RMT	Tunable size and release; scalable manufacturing	Burst release; anti‐PEG immune response (ABC)	MCAO mouse: ↓ infarct volume 35–50%, ↓ IL‐1β and TNF‐α	[173, 183]
Dendrimers	PAMAM, PPI	5–20	Adsorptive‐mediated transcytosis	Precise architecture; multivalent modification	Dose‐dependent cytotoxicity; synthesis cost	MCAO rat: ↑ neuronal survival, ↓ ROS accumulation	[184]
Inorganic nanoparticles	CeO_2_, SPIONs, Au NPs	5–50	Passive diffusion; magnetic guidance; FUS‐enhanced	ROS‐scavenging; imaging‐guided delivery	Long‐term biodistribution and clearance concerns	MCAO mouse: ↓ MDA 40–60%, ↓ infarct volume ∼50%	[185, 186, 187, 188]
Biomimetic nanoparticles	Cell membrane‐coated NPs (platelet, macrophage)	100–200	Homotypic targeting; immune evasion	Superior lesion targeting; inflammation tropism	Scale‐up, GMP, and batch consistency challenges	MCAO mouse: ↓ infarct volume ∼35%, ↓ neutrophil infiltration	[189, 190]

Abbreviations: BBB, blood–brain barrier; FUS, focused ultrasound; LRP1, low‐density lipoprotein receptor‐related protein‐1; MCAO, middle cerebral artery occlusion; mNSS, modified neurological severity score; RMT, receptor‐mediated transcytosis; ROS, reactive oxygen species; SPIONs, superparamagnetic iron oxide nanoparticle; TfR, transferrin receptor.

### 5.2 Active Targeting: Ligand‐Mediated Transport

Active targeting strategies leverage the overexpression of specific receptors on brain endothelial cells to facilitate RMT, thereby achieving substantially enhanced BBB penetration efficiency and brain‐targeting specificity compared with passive approaches [181]. Commonly used BBB‐targeting ligands, their cognate receptors, and representative applications in stroke models are summarized in Table 2.

**TABLE 2:** Representative BBB‐targeting ligands and receptors applied in stroke nanomedicine.

Targeting ligand	Cognate receptor	Transport mechanism	Key advantages	Representative application (model/outcome)	References
Transferrin (Tf)	Transferrin receptor (TfR)	Receptor‐mediated transcytosis	Well‐characterized BBB pathway	MCAO rat, i.v., 1–3 h: ↑ brain accumulation, ↓ infarct volume ∼25%	[181, 191, 192, 193, 194, 195]
Lactoferrin (Lf)	Lactoferrin receptor	Receptor‐mediated transcytosis	Reduced endogenous competition; intrinsic anti‐inflammatory activity	MCAO mouse: superior BBB penetration vs. Tf‐NPs	[196, 197, 198, 199]
TAT peptide	Heparan sulfate proteoglycans	Adsorptive‐mediated transcytosis	Strong BBB penetration capability	MCAO rat: ↑ neuronal uptake, ↓ apoptosis	[184]
Angiopep‐2	LRP1	Receptor‐mediated transcytosis	High transcytosis efficiency; clinical validation	Preclinical stroke models; ANG1005 in CNS tumors	[182, 200]
Dual‐targeting ligands	TfR + lesion‐specific receptors	Combined RMT + lesion binding	Enhanced specificity and retention	MCAO mouse: ↓ infarct ∼40%, ↑ behavioral recovery	[200]

Abbreviations: BBB, blood–brain barrier; LRP1, low‐density lipoprotein receptor‐related protein‐1; MCAO, middle cerebral artery occlusion; RMT, receptor‐mediated transcytosis.

Among the most extensively investigated receptor‐targeting ligands, Tf has emerged as a particularly promising candidate, primarily due to the overexpression of TfR on the luminal surface of brain capillary endothelial cells, which physiologically facilitate RMT of iron‐bound Tf across the BBB [169]. Optimization of anti‐TfR antibody affinity has emerged as a critical consideration; lower‐affinity antibodies demonstrate superior transcytosis efficiency compared with high‐affinity variants that remain trapped in endosomes [191, 192]. Early‐phase clinical investigations of TfR‐targeted systems for glioblastoma have demonstrated safety and feasibility in Phase I trials; however, no Phase II survival data are currently available for TfR–liposomal formulations in this indication, and the clinical evidence base remains limited to preliminary safety and pharmacokinetic assessments [193]. However, several critical challenges limit the translational potential of Tf‐mediated targeting in stroke. First, receptor saturation represents a fundamental constraint: TfR density on brain endothelium is finite (approximately 10,000–50,000 receptors per cell), and competition with endogenous Tf (present at 2–3 mg/mL in plasma) significantly reduces nanoparticle binding efficiency [194]. In ischemic stroke, this competition is further complicated by iron dysregulation and altered TfR expression in the penumbral region, where TfR may be downregulated during acute ischemia and upregulated during reperfusion [195, 201]. Second, stroke‐specific dosing kinetics remain poorly characterized: the optimal dosing window (0–6 h postreperfusion), dose–response relationships, and pharmacokinetic profiles of Tf‐conjugated nanoparticles in the context of BBB disruption heterogeneity have not been systematically investigated [202]. The biphasic pattern of BBB opening may create variable targeting efficiency depending on administration timing, with early administration potentially achieving greater accumulation through both RMT and paracellular leak, while delayed administration may rely primarily on transcytosis through partially restored barrier [203, 204]. Third, the therapeutic cargo capacity of TfR‐targeted systems is constrained by the need to maintain appropriate ligand density for efficient transcytosis while avoiding receptor clustering that triggers lysosomal degradation rather than transcytosis [205]. These limitations underscore the need for stroke‐specific optimization studies examining dose–timing–efficacy relationships and head‐to‐head comparisons with alternative targeting approaches in relevant ischemia–reperfusion models [206].

Lf represents an alternative iron‐binding glycoprotein that interacts with LfRs, which are abundantly expressed on brain capillary endothelial cells. This ligand offers distinct advantages, including reduced competition with endogenous counterparts compared with Tf, as well as intrinsic anti‐inflammatory and neuroprotective properties [196, 197]. Nanoparticles functionalized with Lf have demonstrated superior capacity for BBB transcytosis across a range of preclinical models [198, 199].

Peptide‐based targeting ligands have emerged as versatile and attractive alternatives to protein‐based moieties. Among these, cell‐penetrating peptides such as TAT and penetratin, along with receptor‐targeting peptides like angiopep‐2, exhibit exceptional capabilities for BBB translocation through distinct yet complementary mechanisms [184]. Angiopep‐2, derived from the Kunitz domain of aprotinin, has advanced to clinical trials for paclitaxel delivery (ANG1005) in brain metastases, demonstrating favorable safety profiles and preliminary efficacy signals [182]. Dual‐targeting strategies combining BBB‐penetrating peptides with disease‐specific targeting moieties have demonstrated synergistic improvements in both brain delivery and disease site accumulation [200].

### 5.3 Stimuli‐Responsive Systems

Stimuli‐responsive nanocarriers have emerged as an innovative and promising paradigm in brain‐targeted drug delivery, enabling precise spatiotemporal modulation of BBB penetration and controlled drug release in response to a diverse array of exogenous or endogenous stimuli.

FUS combined with microbubbles has emerged as the most clinically advanced noninvasive technique for transiently disrupting the BBB, leveraging mechanical bioeffects such as acoustic cavitation, microstreaming, and radiation force to enhance localized drug delivery while preserving tissue integrity [207, 208, 209]. Clinical implementation requires careful parameter optimization, including acoustic pressure (typically 0.2–0.6 MPa), pulse repetition frequency, microbubble concentration, and real‐time MRI monitoring for safety [210]. Clinical trials in AD (NCT03671889, NCT04118764) and glioblastoma have demonstrated safety and feasibility, with BBB opening persisting 4–6 h followed by complete restoration [211, 212]. Integration of therapeutic nanoparticles with FUS offers synergistic enhancement of drug delivery, with studies reporting 5–10 fold increases in brain drug concentrations [213, 214].

Magnetic nanoparticles, particularly superparamagnetic iron oxide nanoparticles, exemplify the theranostic paradigm by integrating therapeutic drug delivery with diagnostic MRI contrast enhancement, while simultaneously enabling external magnetic field‐guided targeting to enhance brain accumulation [215]. Practical considerations for magnetic targeting include field gradient strength (typically 50–500 mT/m), magnet positioning, and imaging coregistration for treatment planning [216]. Alternating magnetic fields enable localized hyperthermia through nanoparticle oscillation, potentially triggering drug release from thermosensitive carriers [217]. Recent advances in magnetoelectric nanoparticles offer wireless deep brain stimulation capabilities, expanding applications beyond drug delivery [218].

pH‐sensitive nanomaterials represent a rational design strategy that exploits the well‐characterized pH gradients in pathological brain tissue, including the acidic tumor microenvironment (pH 6.5–6.8) and endosomal compartments (pH 5.0–6.0), to achieve tumor‐selective drug release while minimizing off‐target toxicity through pH‐triggered structural transformations [219, 220]. Design considerations include cleavage kinetics of pH‐sensitive linkages and mechanisms for endosomal escape following cellular uptake [221]. Dual‐stage responsive systems have been engineered to sequentially navigate systemic circulation, cross the BBB, and release cargo in response to disease‐specific pH changes, achieving sophisticated spatiotemporal control over drug delivery [222].

In summary, multifunctional and biomimetic nanoplatforms represent an evolution toward more comprehensive therapeutic designs capable of addressing the multifactorial nature of ischemic stroke. By combining targeting, responsive release, and multimechanistic action, these systems offer conceptual advantages over single‐function carriers. However, their increased structural and biological complexity introduces additional challenges related to reproducibility, safety, and scalability. Balancing functional sophistication with translational feasibility therefore remains a central consideration, leading naturally to a broader discussion of clinical translation and regulatory perspectives.

## 6 Mechanism‐Based Classification of Nanotherapeutics for Stroke

**FIGURE 5:**
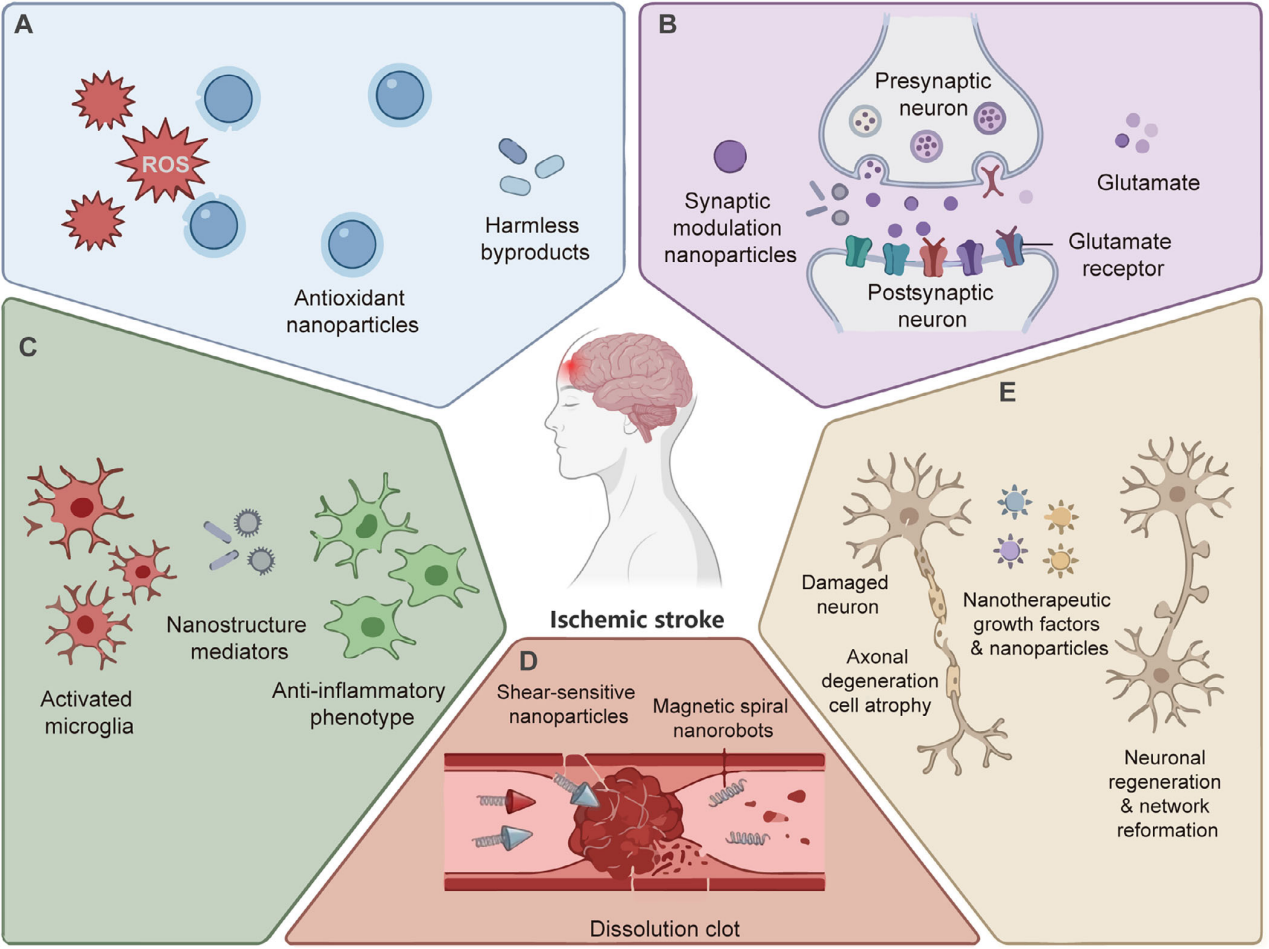
Multifaceted nanotherapeutic strategies for ischemic stroke intervention. The schematic illustrates five key mechanisms of nanoparticle‐mediated stroke treatment centered around an ischemic brain: (A) ROS‐scavenging by antioxidant nanoparticles neutralizing reactive oxygen species into harmless byproducts; (B) glutamate modulation at the synaptic cleft through receptor‐targeted nanoparticles reducing excitotoxicity; (C) anti‐inflammatory effects via nanostructure mediators promoting microglial phenotype conversion from proinflammatory (red) to anti‐inflammatory (green) states; (D) thrombolytic action using shear‐sensitive nanoparticles and magnetic nanorobots for targeted clot dissolution; (E) neuroprotection and regeneration through delivery of growth factors stimulating axonal regrowth and neuronal network reformation.

Ischemic stroke triggers a complex cascade of pathophysiological events, including excitotoxicity, oxidative stress, neuroinflammation, and apoptosis, each presenting distinct therapeutic targets amenable to nanomaterial‐based intervention [223]. The development of nanotherapeutics for stroke has evolved from simple drug carriers to sophisticated platforms designed to address specific pathological mechanisms at optimal time points during stroke progression [224]. Mechanism‐based classification provides a rational framework for understanding how different nanomaterial strategies target specific aspects of stroke pathophysiology, enabling more precise therapeutic design and clinical translation [225]. This approach recognizes that the temporal evolution of ischemic injury—from acute excitotoxicity (minutes to hours) through oxidative stress and inflammation (hours to days) to delayed apoptosis and tissue remodeling (days to weeks)—requires temporally matched therapeutic interventions [185]. Notably, multifunctional and biomimetic nanoplatforms are discussed herein as an integrative design paradigm that spans multiple mechanistic categories, rather than as a standalone therapeutic mechanism. In this section, we systematically examine nanotherapeutics classified by their primary mechanism of action, highlighting representative examples, preclinical efficacy, and translational considerations for each category (Figure 5).

### 6.1 ROS‐Scavenging Nanomaterials

Carbon‐based nanomaterials, particularly fullerenes and their functionalized derivatives, constitute a distinctive class of catalytic ROS‐scavenging agents. The parent fullerene (C_60_), with its highly symmetrical spherical architecture and extensive π‐conjugated system, exhibits broad‐spectrum reactivity toward diverse reactive oxygen and nitrogen species, functioning as a regenerable “radical sponge.” Polyhydroxylated fullerene derivatives (fullerenols, C_60_(OH)*_n_*) have demonstrated remarkable neuroprotective effects in experimental stroke models, reducing infarct volume by 53–81% in middle cerebral artery occlusion (MCAO) studies [186].

Quantitative assessments of ROS‐scavenging efficacy have demonstrated significant reductions in oxidative stress biomarkers following nanomaterial treatment [187]. Cerium oxide nanoparticles (nanoceria), with their unique ability to cycle between Ce^3^^+^ and Ce^4^^+^ oxidation states, have shown 40–60% reduction in malondialdehyde (MDA) levels and 4‐hydroxy‐2‐nonenal (4‐HNE) accumulation in MCAO models. The regenerative catalytic activity of nanoceria, mimicking both superoxide dismutase and catalase [188], provides sustained antioxidant protection exceeding that of small‐molecule antioxidants. Long‐term biodistribution studies indicate preferential accumulation in liver and spleen with gradual clearance [226], though brain retention mechanisms and potential chronic effects require further investigation for clinical translation [227].

### 6.2 Glutamate‐Modulating Nanomaterials

Glutamate excitotoxicity, triggered by excessive activation of N‐methyl‐d‐aspartate receptors and subsequent pathological calcium influx, represents a critical and therapeutically targetable mechanism of neuronal injury in the hyperacute phase of ischemic stroke. This cascade occurs within minutes to hours following arterial occlusion, making it a prime target for early neuroprotective interventions [228].

Therapeutic window optimization is critical for excitotoxicity interventions, as glutamate release peaks within minutes of ischemia onset and receptor‐mediated injury occurs within 1–6 h [229, 230]. NR2B9c–WGA‐NPs targeting the PSD‐95/nNOS interaction have demonstrated efficacy when administered up to 3 h postreperfusion, with improvements in modified neurological severity scores (mNSS) by 2–3 points and reduction in infarct volume by 25–40% at 24 h [231, 232]. Similarly, ZL006‐loaded dual‐targeted liposomes (T7&SHp‐P‐LPs) showed 35% infarct reduction when administered 2 h post‐MCAO, accompanied by improved rotarod performance and Morris water maze outcomes at 7‐day follow‐up [233]. These findings underscore the importance of early intervention and provide guidance for clinical trial design.

### 6.3 Anti‐inflammatory Nanomaterials

Neuroinflammation represents a critical yet complex therapeutic target in ischemic stroke. Characterized by rapid activation of resident microglia and sequential infiltration of peripheral immune cells (neutrophils, monocytes/macrophages, and lymphocytes), the inflammatory response exhibits profound spatiotemporal heterogeneity and functional duality, with both injury‐exacerbating and repair‐promoting components [234, 235]. Recent nanomedicine strategies have focused on modulating microglial polarization to favor neuroprotective M2 phenotypes while suppressing detrimental M1 responses [183].

Quantification of microglial phenotype shifts demonstrates the efficacy of anti‐inflammatory nanotherapeutics. Ma@(MnO_2_+FTY) nanoparticles achieved threefold increase in M2 marker (CD206, Arg‐1) expression and 60% reduction in M1 markers (iNOS, CD86) in the peri‐infarct region at 3 days post‐MCAO [224]. The MnO_2_ component provided additional benefit through catalytic decomposition of H_2_O_2_ to generate O_2_, rescuing hypoxic neurons in the penumbra. M2M@BANPs demonstrated 45% reduction in IL‐1β, 50% reduction in TNF‐α, and 40% reduction in neutrophil infiltration (MPO activity) at 24 h, accompanied by 35% infarct volume reduction and significant improvement in Garcia neurological scores. These comprehensive outcomes support the multitarget efficacy of immunomodulatory nanotherapeutics [189].

### 6.4 Neuroprotective and Neuroregenerative Nanomaterials

Neurotrophic factors, including brain‐derived neurotrophic factor (BDNF), VEGF, nerve growth factor, and GDNF, play essential and complementary roles in promoting neuronal survival, stimulating neurogenesis, facilitating angiogenesis, and enhancing synaptic plasticity following ischemic injury [236]. However, their clinical translation has been limited by poor BBB penetration and short biological half‐lives.

Safety considerations for neurotrophic factor delivery warrant careful attention. Systemic VEGF administration carries risks of aberrant angiogenesis, vascular permeability increase, and potential tumor promotion [237]. BDNF, while generally well tolerated, may induce hyperexcitability and seizure susceptibility at supratherapeutic concentrations [238]. Nanoparticle‐mediated delivery addresses these concerns through localized, temporally controlled release that achieves therapeutic concentrations at target sites while minimizing systemic exposure [239]. Stem cell‐based approaches must consider tumorigenicity risks and potential ectopic tissue formation; engineering stem cells with suicide genes or using nonintegrating gene delivery systems provides additional safety margins [240, 241]. Biodegradable scaffolds with controlled growth factor release kinetics offer spatial and temporal control that may further mitigate safety concerns while enhancing therapeutic efficacy [242].

### 6.5 Multifunctional Nanoplatforms

Given the complex, multifactorial pathophysiology of CNS disorders, multifunctional nanoplatforms that integrate therapeutic, diagnostic, and targeting capabilities to simultaneously address multiple pathological mechanisms represent a transformative paradigm shift in nanomedicine, offering superior efficacy compared with conventional monotherapies [243].

Translational challenges differ substantially between advanced biomimetic/magnetically guided platforms and conventional carriers. Cell membrane‐camouflaged nanoparticles face critical hurdles: (1) membrane sourcing—autologous preparation is patient‐specific and costly, while allogeneic sources (cell lines vs. primary cells) raise immunogenicity and standardization concerns [190, 244]; (2) batch‐to‐batch variability in membrane protein composition, orientation, and density complicates quality control and GMP compliance [245]; (3) immunogenicity assessment requires extensive preclinical evaluation of antimembrane antibody responses; (4) scale‐up from laboratory (mg) to clinical (kg) quantities demands validated continuous‐flow coating processes [246]; (5) release testing must characterize both drug liberation kinetics and membrane integrity over shelf‐life. Magnetically guided systems additionally require specialized equipment for field generation and real‐time imaging coregistration, limiting deployment to specialized centers. In contrast, polymeric PLGA nanoparticles and liposomal formulations benefit from decades of manufacturing experience, established analytical methods, well‐characterized degradation pathways, and multiple United States Food and Drug Administration‐approved products (Doxil, Onpattro) providing regulatory precedent. Consequently, liposomal and polymeric carriers are most likely to reach clinic first for BBB‐targeted stroke therapeutics due to superior manufacturability, reproducibility, and regulatory familiarity [6]. Biomimetic platforms, despite superior preclinical targeting efficacy, will likely require substantial additional time to resolve manufacturing and regulatory challenges before pivotal trials [247].

In conclusion, despite substantial advances in nanomedicine‐based strategies for stroke, successful clinical translation remains contingent upon addressing regulatory, manufacturing, and model‐relevance challenges. Progress will likely depend on standardized characterization, realistic preclinical evaluation, and alignment with established clinical workflows. By integrating mechanistic insight with translational discipline, future nanotherapeutic development can move beyond proof‐of‐concept toward clinically actionable solutions. Collectively, the perspectives outlined herein aim to guide the field toward more robust, reproducible, and ultimately translatable nanomedicine approaches for ischemic stroke.

## 7 Preclinical Studies and Clinical Translation

**FIGURE 6:**
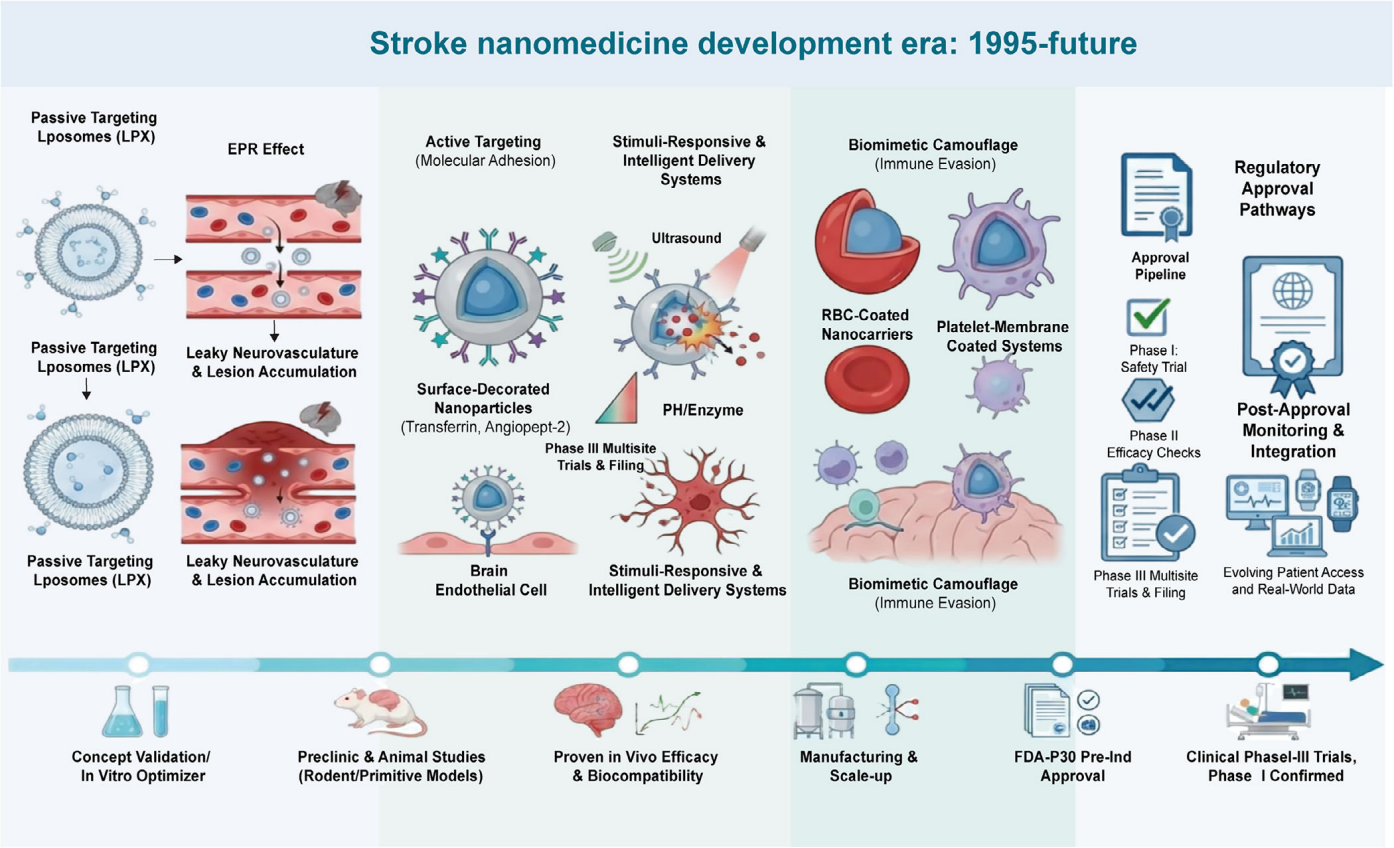
Timeline of key developments in stroke nanomedicine (1995–2024). This timeline illustrates the major milestones in the evolution of nanomedicine for ischemic stroke, from early liposomal formulations and the discovery of the EPR effect through the development of active targeting strategies, stimuli‐responsive systems, and biomimetic platforms. Key regulatory and clinical translation events are highlighted, including US FDA approvals of related nanomedicines and initiation of clinical trials for BBB‐targeted delivery systems. The timeline contextualizes current research within the broader historical trajectory of the field.

The translation of nanotherapeutics from laboratory discoveries to clinical applications represents a critical bottleneck in the field. This section summarizes key preclinical findings and examines the current landscape of clinical trials investigating BBB‐targeted nanomedicines, while identifying gaps between preclinical promise and clinical reality (Figure 6).

### 7.1 Summary of Preclinical Animal Studies

Extensive preclinical studies have demonstrated the therapeutic potential of diverse nanomaterial platforms in animal models of neurological diseases. MCAO models in rodents remain the most commonly employed stroke models, though variations in occlusion duration (30 min to permanent), reperfusion protocols, and outcome assessment timing complicate cross‐study comparisons [248, 249]. Key findings include: (1) cerium oxide nanoparticles reduced infarct volume by 40–60% with improved neurological scores when administered within 4 h of reperfusion [250]; (2) biomimetic platelet membrane‐coated nanoparticles demonstrated targeted thrombus accumulation with 50–70% clot dissolution rates [251, 252]; (3) BDNF‐loaded nanoparticles enhanced neurogenesis markers (BrdU+/DCX+ cells) by twofold to threefold in the subventricular zone at 14 days poststroke [253, 254]. However, most studies employ young, healthy animals with single comorbidity factors, limiting translational relevance to the typically elderly, comorbid patient population. Representative preclinical and clinical studies investigating BBB‐targeted nanotherapeutics for ischemic stroke over the past 5 years are detailed in Table 3.

**TABLE 3:** Representative preclinical and clinical studies of BBB‐targeted nanotherapeutics (2020–2024).

Therapeutic mechanism	Nanoplatform	Payload	Model/timing	Key quantitative outcomes	Translational note	References
BBB opening (clinical)	FUS + microbubbles	—	AD/GBM trials	Reversible BBB opening (4–6 h)	Phase I–II, safe	[211, 212, 213]
ROS‐scavenging	Cerium oxide nanoparticles	—	MCAO mouse, ≤4 h	↓ infarct volume 40–60%; ↓ MDA, 4‐HNE	Long‐term safety under evaluation	[183, 185, 186, 187, 188, 226, 227, 228, 229, 230, 231, 232, 233, 234, 235]
Glutamate modulation	NR2B9c‐WGA‐NPs	PSD‐95/nNOS inhibitor	MCAO rat, ≤3 h	↓ infarct volume 25–40%; ↑ mNSS	Narrow therapeutic window	[230, 231]
Anti‐inflammatory modulation	MnO_2_ + fingolimod NPs	FTY720	MCAO mouse, 2 h	↑ M2/M1 ratio ×3; ↓ IL‐1β ∼45%	Multifunctional benefit	[183]
Biomimetic immunomodulation	M2 macrophage membrane‐coated NPs	Anti‐inflammatory drug	MCAO mouse	↓ infarct volume ∼35%; ↓ neutrophil infiltration	Scale‐up challenge	[189]

Abbreviations: AD, Alzheimer's disease; FUS, focused ultrasound; GBM, glioblastoma multiforme; MCAO, middle cerebral artery occlusion; MDA, malondialdehyde; mNSS, modified neurological severity score; ROS, reactive oxygen species.

### 7.2 Current Clinical Trials and Translational Gaps

Despite decades of promising preclinical data, clinical translation of stroke nanomedicines remains limited. Current clinical trials investigating BBB‐targeted delivery systems include: (1) ANG1005 (angiopep‐2‐conjugated paclitaxel) for brain metastases (NCT03613181, Phase III) [255, 256]; (2) FUS BBB opening with microbubbles in AD (NCT03671889, Phase II) and glioblastoma (NCT04063514, Phase I/II); (3) Magnetic resonance‐guided FUS for trastuzumab delivery in HER2+ breast cancer brain metastases (NCT03714243, Phase I) [257]. Notably, no nanoparticle‐based therapeutics have reached pivotal Phase III trials specifically for ischemic stroke, reflecting the translational challenges unique to this indication, including narrow therapeutic windows, heterogeneous patient populations, and complex outcome measures.

### 7.3 Reasons for Translational Gaps

Several factors contribute to the disconnect between preclinical success and clinical translation in stroke nanomedicine: (1) Animal model limitations: young, healthy rodents with controlled, predictable strokes poorly represent elderly patients with comorbidities and variable stroke presentations [258]; (2) outcome measure discordance: preclinical studies emphasize infarct volume reduction, while clinical trials prioritize functional outcomes assessed at 90 days [259, 260]; (3) treatment timing: most preclinical studies administer treatments within 1–3 h of reperfusion, while clinical scenarios often involve delayed presentation [261]; (4) dose scaling: allometric scaling of nanoparticle doses from rodents to humans remains poorly established; (5) heterogeneity: the pathophysiology of human stroke varies considerably with etiology, location, and severity, challenging the one‐size‐fits‐all therapeutic approach [262]. To enhance translational relevance, future preclinical studies should prioritize: (i) aged animals (18–24 months) with induced comorbidities including streptozotocin‐induced diabetes and angiotensin II‐induced hypertension; (ii) delayed treatment windows (3–6 h postonset) reflecting realistic clinical presentation; (iii) combination protocols with mechanical thrombectomy and/or tissue plasminogen activator (tPA) to model contemporary standard‐of‐care; (iv) imaging biomarkers including DCE‐MRI permeability mapping (Ktrans) and perfusion‐weighted imaging for patient stratification; and (v) serum biomarkers such as IL‐6 and MMP‐9 for patient selection and therapeutic response monitoring [263].

## 8 Conclusions and Future Perspectives

The BBB remains a defining factor in both the pathophysiology of neurological diseases and the success or failure of therapeutic intervention. In this review, we have systematically summarized the current understanding of BBB structure and function, its dynamic alterations in major neurological disorders, and recent advances in BBB‐targeted drug delivery strategies. By organizing nanotherapeutics according to their primary mechanisms of action rather than carrier type, this review provides a coherent framework for understanding how delivery systems may be matched to distinct pathological processes and therapeutic time windows.

Although nanomaterial‐based approaches have demonstrated encouraging results in preclinical studies, their clinical translation has been limited. Key challenges include incomplete characterization of long‐term safety and immunogenicity, uncertainty regarding in vivo degradation and clearance, and off‐target accumulation in peripheral organs. In addition, the growing complexity of multifunctional and biomimetic nanoplatforms poses practical challenges for large‐scale manufacturing, batch‐to‐batch reproducibility, and regulatory evaluation. These factors, together with differences between experimental models and clinical reality, likely contribute to the persistent gap between preclinical efficacy and clinical success.

Future efforts in BBB‐targeted drug delivery should emphasize translational robustness and clinical relevance. The use of biodegradable and well‐characterized nanocarriers, standardized physicochemical and biological evaluation protocols, and disease‐relevant experimental models will be essential to improve reproducibility and predictive value. Human‐relevant BBB models, advanced imaging techniques, and biomarker‐informed patient stratification may further support rational therapeutic development while minimizing unnecessary complexity.

In conclusion, BBB‐targeted nanomedicine represents an important and evolving area of research with clear therapeutic potential. Continued progress will depend on integrating mechanistic insight with practical considerations of safety, manufacturability, and regulatory feasibility. A balanced and clinically grounded approach is likely to be most effective in advancing BBB‐focused delivery strategies toward meaningful clinical application.

## Author Contributions

Conceptualization: Y.M., H.G., and W.M. Writing – original draft preparation: Y.H., M.Q., and L.Y. Writing – review and editing: Y.L., L.H., J.H., M.S., and H.Y. Visualization: Y.H. and M.Q. Supervision: Y.M., H.G., and W.M. Funding acquisition: Y.M. All authors have read and agreed to the published version of the manuscript.

## Conflicts of Interest

The authors declare no conflicts of interest.

## Ethics Statement

The authors have nothing to report.

## Data Availability

The authors have nothing to report.
